# Who Has a Stronger Commitment to Teaching: A Latent Profile Analysis Based on Preservice Teachers’ Learning Experiences in Teacher Education Courses

**DOI:** 10.3390/bs16071157

**Published:** 2026-07-09

**Authors:** Peili Yuan, Weiran Wu, Nairui Yan, Yuze Chen, Huan Song

**Affiliations:** 1 Center for Teacher Education Research, Beijing Normal University, Beijing 100875, China; yuanpeili@mail.bnu.edu.cn; 2 College of Teacher Education, Southwest University, Chongqing 400715, China; 3 College of Teacher Education, South China Normal University, Guangzhou 510631, China; 4 School of Education, Tsinghua University, Beijing 100084, China

**Keywords:** preservice teachers, course learning experiences, commitment to teaching, teacher education, latent profile analysis, teacher self-efficacy

## Abstract

Enhancing preservice teachers’ commitment to teaching is of great significance for improving the stability and sustainable development of the teaching workforce. Teacher education courses constitute a core component of preservice teacher preparation, and preservice teachers’ learning experiences in these courses may be closely associated with their commitment to teaching. Based on survey data from 26,110 preservice teachers at local normal universities in China, this study treated preservice teachers’ learning experiences in teacher education courses as the focal independent variable and commitment to teaching as the dependent variable. Latent profile analysis was used to identify profiles of course learning experiences, and between-profile differences in commitment to teaching were then examined. Teacher self-efficacy was further tested as a mediator in the association between course learning experiences and commitment to teaching within each profile. Three profiles were identified: a comprehensively positive profile, characterized by high scores across good teaching, clear course goals, appropriate assessment, and appropriate workload; a mixed-experience profile, characterized by relatively positive perceptions of good teaching and clear course goals but lower evaluations of assessment and workload; and a moderate-experience profile, characterized by moderate scores across the four dimensions. Significant differences were found in commitment to teaching across the three profiles. Preservice teachers in the comprehensively positive profile reported the highest level of commitment to teaching, followed by those in the mixed-experience profile, while those in the moderate-experience profile reported the lowest level. In addition, mediation analysis showed that teacher self-efficacy played different roles across profiles. Based on these findings, this study proposes directions for reforming teacher education courses, with the aim of improving the quality of preservice teacher preparation and fostering preservice teachers’ commitment to teaching.

## 1. Introduction

In recent years, the growing tendency among preservice teachers not to enter the education sector after graduation has attracted increasing attention from governments and scholars worldwide (Ingersoll & Tran, 2023). Commitment to teaching, as a motivational psychological state in which individuals decide to enter the teaching profession and prepare themselves for it, is reflected in preservice teachers’ identification with the teaching profession and their willingness to become members of the teaching workforce in the future (Klassen & Chiu, 2011). Preservice teachers’ commitment to teaching is closely associated with whether they will choose and remain in the teaching profession in the future (Chapman, 1983; Rots et al., 2010), and serves as an important indicator for predicting their professional loyalty, job performance, intention to stay or leave, and work engagement (Chapman & Green, 1986). Currently, the teaching commitment of preservice teachers in China faces notable challenges. High rates of mobility and contract breach have been reported among government-funded preservice teachers (Li & Zhang, 2022; Wang, 2019), while many graduates from teacher education programs do not view teaching as their preferred career path (Song et al., 2018). Therefore, attending to and enhancing preservice teachers’ commitment to teaching is of great value for ensuring the stability and sustainable development of the teaching workforce.

At present, teacher education reform in China has entered a more complex and challenging stage, and teacher education course reform has become a key lever for improving the quality of teacher education. Previous research has shown that preservice teacher preparation, especially adequate preparation in pedagogical methods, practice teaching, classroom observation, and teaching feedback, is closely related to beginning teachers’ retention in the profession (Ingersoll et al., 2012). Since the issuance of the Opinions of the Ministry of Education on Vigorously Promoting Teacher Education Curriculum Reform in 2011, a series of policy documents have been introduced, emphasizing the need to deepen teacher education reform and comprehensively improve the quality of teacher preparation by innovating curriculum concepts, optimizing curriculum structures, and reforming curriculum content (Ministry of Education of the People’s Republic of China, 2011). However, in practice, reforms in local normal universities still have certain limitations. They have focused more on adjusting talent cultivation models and organizational structures, while giving insufficient attention to, and making limited breakthroughs in, curriculum reform. As a result, a gap remains between policy goals and actual outcomes (Dong et al., 2020). The Outline of the Plan for Building China into a Leading Country in Education (2024–2035) clearly states the need to “improve the quality of teacher education” (Central Committee of the Communist Party of China & State Council of the People’s Republic of China, 2025). As the most important stakeholders in teacher education, preservice teachers have the most direct experience of the quality of teacher education courses. Therefore, attending to and improving preservice teachers’ course learning experiences is of great significance for enhancing the quality of teacher education. Course learning experiences are often personal and complex. Existing variable-centered approaches tend to examine course satisfaction at the group level as a whole, which may overlook the heterogeneity in how preservice teachers, as individual learners, perceive course quality. Therefore, this study adopts a person-centered approach to identify profiles of preservice teachers’ learning experiences in teacher education courses and to examine their relationship with commitment to teaching. In doing so, it aims to provide insights into teacher education course reform and, ultimately, to improve the quality of teacher education.

## 2. Literature Review

For preservice teachers, commitment to teaching reflects the strength of their willingness to choose the teaching profession and to devote themselves firmly to it. The social learning theory of career decision making suggests that individuals’ career decisions are shaped by genetic endowments, environmental conditions, learning experiences, and task-approach skills. It further emphasizes that individuals gradually refine their career interests and professional competencies through continuous self-observation and self-evaluation during learning and training (Krumboltz et al., 1976; Krumboltz & Vosvick, 1996). Teacher education plays an important role in shaping preservice teachers’ attitudes toward the teaching profession, motivation to teach, and career choices (Moses et al., 2021; Neugebauer, 2015). Drawing on the social learning theory of career decision making, Rots and Aelterman (2008, 2009) proposed and validated a theoretical model explaining the factors that influence preservice teachers’ career choices. They argued that preservice teachers’ experiences in teacher education programs directly affect their commitment to teaching. Teacher education programs may also shape preservice teachers’ cognitive and affective orientations toward the teaching profession by enhancing teacher self-efficacy, thereby influencing their commitment to teaching (Rots & Aelterman, 2008, 2009). In this sense, teacher self-efficacy may serve as an important psychological pathway through which learning experiences in teacher education courses are associated with preservice teachers’ commitment to teaching. Based on this, the present study focuses on preservice teachers’ learning experiences in education-related courses within teacher education programs and examines whether teacher self-efficacy mediates the association between course learning experiences and commitment to teaching across different latent profiles.

### 2.1. Preservice Teachers’ Commitment to Teaching

Commitment refers to making a definite choice and personally investing in it. Commitment to teaching reflects teachers’ perceived importance of, and emotional attachment to, the teaching profession (Lamote & Engels, 2010). Upon entering university, preservice teachers usually make an initial and tentative career choice. As they progress through teacher education programs, they gradually determine whether this choice was appropriate and whether the teaching profession is worthy of long-term personal investment. Some scholars further point out that preservice teachers’ commitment to teaching refers to a motivational psychological state in which they decide to enter the field of education and teaching and prepare themselves accordingly. It includes preparation for future development in education and teaching, beliefs about their ability to carry out teaching fully and effectively, expectations regarding the work of teaching itself, personal affective and cognitive identification, and an accurate estimation of the time and effort required to adapt to and sustain a teaching career (Chesnut, 2017). Existing research on teacher commitment has mainly focused on in-service teachers. Scholars have examined teachers’ levels of commitment to teaching in different national contexts, the factors influencing such commitment, and its role in teachers’ professional development, whereas relatively little attention has been paid to preservice teachers’ commitment to teaching (Sinclair, 2008; Troman & Raggl, 2008). Because preservice teachers’ professional commitment is closely related to issues of career choice, is characterized by considerable uncertainty, and involves multiple aspects of preservice education, its structural measurement tends to focus on indicators related to professional development. Rikard proposed that preservice teachers’ commitment to teaching comprises four dimensions: expectations regarding what students can achieve in their learning, namely commitment to student learning; a reasonable anticipation of the responsibilities required of teachers, namely commitment to teaching work; confidence in one’s ability to effectively influence students’ learning through teaching, namely belief in teaching self-efficacy; and commitment to the school as an organization (Rikard, 1999). Moses et al. (2017) suggested that preservice teachers’ professional commitment can be divided into three dimensions: commitment to teaching, motivation to enter the teaching profession, and perceptions of teaching and the teaching environment. Based on their survey findings, they further identified four types of preservice teachers in terms of commitment to teaching: committed enthusiasts, committed compromisers, undecided preservice teachers, and uncommitted preservice teachers. Cheng and Zhao (2023) used a mixed-methods approach to examine the influence of a teaching competition workshop, as a professional learning community, on Chinese preservice teachers’ commitment to teaching. They found that participation in the workshop had a significant positive effect on preservice teachers’ professional commitment. The above studies provide an important foundation for understanding and measuring preservice teachers’ commitment to teaching.

### 2.2. Preservice Teachers’ Learning Experiences in Teacher Education Courses and Commitment to Teaching

Course learning experience refers to the process through which students gradually develop emotional responses and construct meaning based on their perceptions and deeper understanding of the courses they take. It is regarded as an important indicator of course quality (Ramsden, 1991). It mainly includes four dimensions: the clarity of course goals, good teaching, the appropriateness of assessment methods, and the reasonableness of workload (Wilson et al., 1997). Heinz (2015) found that preservice teachers’ perceptions of education and the teaching profession are jointly shaped by their prior experiences and teacher education programs, which in turn influence their commitment to teaching. Empirical studies have shown that positive course learning experiences contribute to the coordinated development of students’ cognitive and non-cognitive competencies. When preservice teachers perceive a supportive learning environment characterized by clear goals and positive classroom interactions, their learning outcomes tend to improve significantly (Furlong et al., 2008). Preservice teachers’ experiences in teacher education programs can be divided into university-based learning experiences in teacher education courses and school-based practicum experiences. Previous research on student teachers’ initial teacher preparation experiences has shown that these experiences involve core themes such as changing professional identities, relationships with others, and the perceived relevance of course provision, and that they may vary according to preparation routes, age, and prior expectations (Hobson et al., 2008). Moses and colleagues examined various forms of university-based experiences that influence preservice teachers’ commitment to teaching (Moses et al., 2021). Another important line of research has focused on preservice teachers’ perceptions of course requirements, including academic workload, level of difficulty, and assessment standards. For example, studies have shown that when preservice teachers perceive their courses as overly difficult or academically demanding, their willingness to continue in teacher education and enter the profession tends to decrease (Chambers et al., 2010; Roness & Smith, 2010). However, Sinclair’s study found that coursework with an appropriate level of challenge can positively contribute to preservice teachers’ commitment to teaching (Sinclair, 2008). Beyond course structure, teacher educators’ pedagogical approaches and instructional practices also play a direct role in shaping preservice teachers’ teaching beliefs and professional identity (Lee & Yuan, 2014; Tang et al., 2014). Some studies have shown that teacher educators’ role-modeling effect does not have a significant influence on commitment to teaching. Instead, a lack of peer assistance and mentor support may lead to motivational decline and lower levels of commitment (Chambers et al., 2010; Sinclair, 2008). Workload and teacher educators’ teaching practices are both important dimensions of course learning experiences. Therefore, further investigation is needed into how Chinese preservice teachers’ learning experiences in teacher education courses are associated with their commitment to teaching. Current research on Chinese preservice teachers’ learning experiences has mainly focused on online courses or learning experiences in specific subject areas, whereas studies on education-related courses in teacher education have been largely theoretical. Therefore, adopting a person-centered perspective, this study conducts a latent profile analysis (LPA) based on four dimensions of course learning experiences—good teaching, clarity of goals, appropriateness of assessment, and appropriateness of workload—to reveal the complexity and heterogeneity of preservice teachers’ learning experiences. It further examines whether preservice teachers with different profiles of course learning experiences differ significantly in their commitment to teaching.

### 2.3. Teacher Self-Efficacy and Commitment to Teaching

Teacher self-efficacy mainly comprises efficacy in three domains: student engagement, instructional strategies, and classroom management (Tschannen-Moran & Woolfolk Hoy, 2001). It reflects preservice teachers’ confidence in their teaching capabilities and directly influences how they approach teaching tasks and their willingness to remain in the teaching profession over the long term. Preservice teachers may develop or lose their sense of teacher self-efficacy during teacher education, which in turn may influence their commitment to teaching. Studies have shown that higher levels of teacher self-efficacy are associated with stronger commitment to teaching (Coladarci, 1992; Ware & Kitsantas, 2007). Individuals tend to avoid activities, environments, or work demands that they perceive as exceeding their capabilities. Conversely, they are more willing to engage in tasks they believe fall within their capacity. Therefore, if individuals perceive a profession as beyond their personal capabilities before making a career choice, they may develop apprehension toward it. Similarly, if they encounter tasks that exceed their perceived competence during their work, they may be inclined to withdraw or avoid them (Chesnut, 2017). Studies have found that teacher guidance not only provides positive role models but also helps preservice teachers develop teaching skills, improve communication and feedback practices, and enhance their teacher self-efficacy (Hobson, 2003; Rots et al., 2010). In addition, a number of studies have indicated that preservice teachers’ perceived teaching preparedness is significantly and positively associated with their commitment to teaching (Rots & Aelterman, 2009; Rots et al., 2014). However, some studies have also found that preservice teachers’ perceived preparedness is not significantly associated with their decision to enter the teaching profession (Canrinus & Fokkens-Bruinsma, 2014; Taylor & Frankenberg, 2009). Therefore, further investigation is needed to examine the relationship between teacher self-efficacy and commitment to teaching in the Chinese context. Chesnut and Burley (2015) found that negatively oriented measures of commitment, such as burnout and intention to leave, were more strongly associated with teacher self-efficacy than positively oriented measures, such as career persistence and job attachment. Based on the above analysis, this study addresses the following research questions:

(1) What distinct latent profiles of course learning experiences can be identified among Chinese preservice teachers?
(2) Are there significant differences in teacher self-efficacy and commitment to teaching across these latent profiles?
(3) Does the mediating role of teacher self-efficacy in the association between course learning experiences and commitment to teaching differ across the identified latent profiles?

## 3. Method

### 3.1. Participants

The data for this study were obtained from the Chinese Teacher Education Quality Database (TEQD), a long-term follow-up study tracking a cohort of undergraduate preservice teachers to explore issues related to preservice teacher learning and teacher education quality in China. Given the relatively small proportion of preservice teachers within the overall student population in China and the need for a cost-effective yet representative sample, the TEQD employed a purposive sampling method (Campbell et al., 2020). Participants were drawn from 17 local normal universities and teacher education colleges located in 17 provinces or autonomous regions across eastern, central, and western China (see Table 1 for sample distribution). The TEQD conducted its baseline surveys and interviews in the summer of 2022, followed by revisions to the survey instruments and the first wave of data collection in the spring and summer of 2023. In 2024, an additional survey was administered to senior students entering their internship phase at selected institutions. The survey was conducted online via the Teacher Training and Development Data Platform, with a total of 46,745 preservice teachers from the 2019, 2020, 2021, and 2022 cohorts participating in the first wave of data collection. Since the present study focuses on student teachers’ course experience, and preservice teachers typically complete most of their education-related coursework during their second and third years, only data from sophomore and junior student teachers were analyzed. After data cleaning, which involved excluding responses with missing values or completion times under 10 min, a total of 26,110 valid responses were included in the final analysis.

### 3.2. Measures

#### 3.2.1. Preservice Teachers’ Course Learning Experiences

The Course Learning Experiences Scale used in this study was adapted from the Course Experience Questionnaire developed by Ginns et al. (2007). To ensure the scales’ content validity and contextual appropriateness, experts in teacher education were consulted to evaluate the questionnaire’ s relevance and clarity. Based on the experts’ suggestions, item statements and specific wording were revised to ensure that the scales were appropriate for the context of preservice teacher education in China. The adapted scale retained the same dimensional structure as the original instrument, comprising four dimensions: (1) good teaching (GT): students’ perceptions of whether instructors made the course content engaging, stimulated their learning motivation, explained knowledge clearly, and aroused their interest; (2) clear course goals scale (CCG): whether students clearly understood course expectations, learning objectives, and assignment standards; (3) appropriate assessment scale (AA): whether assessment emphasized understanding and analysis rather than rote memorization or mere recall of factual knowledge; and (4) appropriate workload scale (AW): students’ perceptions of course pressure, academic burden, and task load. It included 14 items, rated on a five-point Likert scale. One item in clear course goals and all items in the appropriate assessment and appropriate workload dimensions were negatively worded and therefore reverse-coded, so that higher scores consistently indicated more positive course learning experiences. Confirmatory factor analysis indicated that the scale demonstrated acceptable structural validity (CFI = 0.931, TLI = 0.921, GFI = 0.934, RMSEA = 0.052). The Cronbach’s alpha coefficient was 0.836, indicating good reliability.

#### 3.2.2. Teacher Self-Efficacy

The scale was based on the Teacher Sense of Efficacy Scale (Tschannen-Moran & Woolfolk Hoy, 2001) and was contextually adapted according to experts’ suggestions. The scale comprised 12 items across three dimensions: student engagement, instructional strategies, and classroom management. Items were rated on a five-point Likert scale, ranging from 1 = “not at all confident” to 5 = “very confident.” Confirmatory factor analysis indicated good structural validity (CFI = 0.972, TLI = 0.931, GFI = 0.942, RMSEA = 0.060). The Cronbach’s alpha coefficient was 0.952, indicating good reliability.

#### 3.2.3. Commitment to Teaching

Commitment to teaching was measured using the commitment to teaching subscale of the Teacher Professional Identity Scale developed by Wong and Liu (2024). Respondents were asked to indicate the extent to which they agreed with each statement, such as “I would leave teaching for another profession if I could.” Responses were rated on a five-point Likert scale ranging from 1 (completely disagree) to 5 (completely agree). Negatively worded items were reverse-coded so that higher scores indicated stronger commitment to teaching. Confirmatory factor analysis indicated that the scale demonstrated acceptable structural validity (CFI = 0.967, TLI = 0.942, GFI = 0.922, RMSEA = 0.064). The Cronbach’s alpha coefficient was 0.914, indicating good reliability.

### 3.3. Procedures

To ensure ethical compliance and participant confidentiality, all preservice teachers were informed that their responses would be anonymous and used solely for research purposes. When accessing the online survey platform, developed by the research team, participants were first presented with an informed consent statement on the homepage, outlining the background and purpose of the study, as well as their right to withdraw at any time—without consequence. Only after acknowledging this information did they proceed to complete the survey. Upon submission, participants were eligible for a lottery-based cash compensation, with varying amounts randomly allocated through the survey platform.

### 3.4. Data Analysis

Data were analyzed using Mplus 8.0 and SPSS 24.0. The analytic procedures were as follows. First, latent profile analysis was conducted to identify profiles of Chinese preservice teachers’ learning experiences in teacher education courses. Second, following Vermunt (2010), the improved Bolck–Croon–Hagenaars (BCH) method was used to examine whether preservice teachers with different profiles differed significantly in commitment to teaching and teacher self-efficacy. Third, mediation analyses were conducted separately within each latent profile using Model 4 of Hayes’ PROCESS macro for SPSS to examine whether teacher self-efficacy mediated the association between course learning experiences and commitment to teaching.

## 4. Results

### 4.1. Identifying Latent Profiles of Preservice Teachers’ Course Learning Experiences

Table 2 presents the descriptive statistics for the study variables. The mean score for overall course learning experiences was 3.316 (SD = 0.579). Among the four dimensions of course learning experiences, good teaching had the highest mean score (M = 3.825, SD = 0.773), whereas appropriate assessment had the lowest mean score (M = 2.861, SD = 0.964). The mean scores for teacher self-efficacy and commitment to teaching were 3.915 (SD = 0.618) and 3.660 (SD = 0.643), respectively.

Latent profile analysis (LPA) is an iterative model-testing procedure (Tein et al., 2013), in which the optimal number of profiles is determined through model comparison (Marsh et al., 2009). Model selection was based on multiple fit indices. Lower values of LL, AIC, BIC, and sample-size-adjusted BIC (SSA-BIC) indicate better model fit. Entropy was also considered in model selection, with values closer to 1.0 indicating higher classification quality. The LMR and BLRT tests were expected to reach statistical significance (*p* < 0.05).

LPA fit indices do not have strict cutoff criteria (Gabriel et al., 2015). In addition to statistical fit indices, the theoretical interpretability of the latent profiles was also considered an important criterion for model selection (Foti et al., 2012). As noted by Dahling et al. (2017), even when fit indices provide slight support for alternative solutions, researchers should give priority to parsimony and interpretability. Parsimony requires selecting a model with fewer profiles to maintain analytic simplicity, whereas interpretability ensures that the identified profiles can be meaningfully understood within the theoretical framework.

As shown in Table 3, fit indices improved as the number of profiles increased from two to six. This pattern is common in large-sample LPA studies, where information criteria and likelihood-ratio tests may continue to favor more complex models even when additional profiles provide limited substantive value. Therefore, the final model was not selected solely on the basis of the lowest information criteria. Instead, we further examined whether the additional profiles in the four-, five-, and six-profile solutions represented substantively distinct configurations of course learning experiences.

Considering the statistical fit indices, classification quality, parsimony, and theoretical interpretability, the three-profile solution was ultimately retained as the optimal model for the present study. As shown in Table 3, compared with the two-profile solution, the three-profile solution yielded lower LL, AIC, BIC, and SSA-BIC values, and both the LMR and BLRT tests reached statistical significance. By contrast, inspection of the four-, five-, and six-profile solutions indicated that the additional profiles mainly subdivided the broader patterns identified in the three-profile solution into finer-level differences, rather than yielding additional qualitatively distinct and theoretically meaningful configurations. Although these more complex solutions showed lower information criteria and, in some cases, higher entropy, they increased classification complexity without substantially improving the conceptual clarity or practical usefulness of the profile structure. Retaining a more complex solution would also make subsequent BCH comparisons and profile-specific mediation analyses more fragmented and less interpretable.

**Figure 1:**
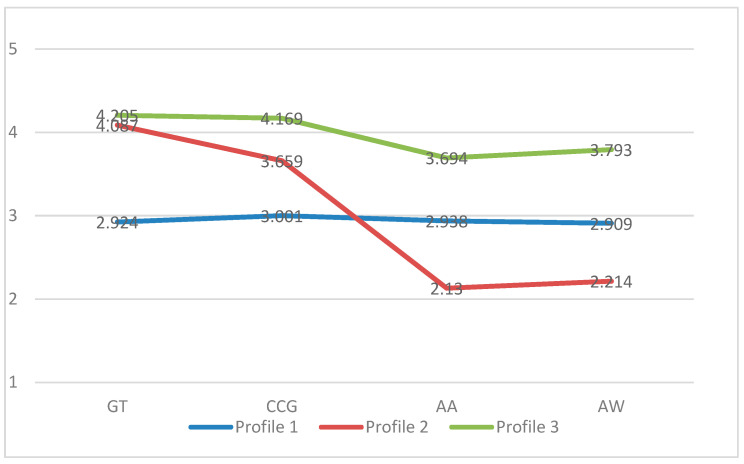
Three latent profiles of preservice teachers’ course learning experiences. Note. GT = good teaching; CCG = clear course goals; AA = appropriate assessment; AW = appropriate workload.

Accordingly, the three-profile solution was selected as the optimal model because it achieved the best balance among statistical adequacy, parsimony, classification clarity, and substantive interpretability. This solution provided a stable and theoretically meaningful basis for examining differences in commitment to teaching and teacher self-efficacy across profiles, as well as for conducting subsequent profile-specific mediation analyses (see Figure 1).

As shown in Figure 1 and Table 4, the three latent profiles of course learning experiences among preservice teachers at local normal universities differed significantly across all four dimensions, with clearly distinguishable characteristics.

Moderate-experience profile (Profile 1: n = 6640; M_GT_ = 2.92, M_CCG_ = 3.00, M_AA_ = 2.94, M_AW_ = 2.91) accounted for 25.4% of the total sample. Preservice teachers in this group reported scores around the scale midpoint, indicating that their overall learning experiences in teacher education courses were at a moderate level.

Mixed-experience profile (Profile 2: n = 10,674; M_GT_ = 4.09, M_CCG_ = 3.66, M_AA_ = 2.13, M_AW_ = 2.21) represented the largest subgroup, accounting for 40.9% of the total sample. Preservice teachers in this profile reported above-average scores for the quality of teacher educators’ teaching and the clarity of course goals, but significantly lower scores for workload and assessment methods. This suggests that students in this profile were dissatisfied with the course workload and assessment practices.

Comprehensively positive profile (Profile 3: n = 8796; M_GT_ = 4.21, M_CCG_ = 4.17, M_AA_ = 3.69, M_AW_ = 3.79) included 33.7% of the preservice teachers. Preservice teachers in this group reported above-average learning experiences across all dimensions, indicating an overall positive attitude toward their learning experiences in teacher education courses.

### 4.2. Differences in Commitment to Teaching and Teacher Self-Efficacy Across Profiles of Preservice Teachers

The improved BCH method was used to examine differences in commitment to teaching and teacher self-efficacy across the identified profiles of preservice teachers. As shown in Table 5, the results indicated significant differences in both commitment to teaching and teacher self-efficacy across the three profiles of preservice teachers. Among them, the comprehensively positive profile had the highest scores for both teacher self-efficacy and commitment to teaching, indicating that preservice teachers in this group not only possessed a strong sense of teacher self-efficacy but also held a firm commitment to entering the teaching profession. Preservice teachers in the mixed-experience profile reported a moderate-to-high level of commitment to teaching and a relatively high level of teacher self-efficacy, although both scores were lower than those of the comprehensively positive profile. By contrast, those in the moderate-experience profile reported the lowest levels of teacher self-efficacy and commitment to teaching. To further assess the practical significance of these differences, pairwise Cohen’s d effect sizes were reported in Table 6. The effect-size results showed large differences between the moderate-experience profile and the other two profiles in both commitment to teaching and teacher self-efficacy. For commitment to teaching, the difference between the mixed-experience profile and the comprehensively positive profile was small to moderate in magnitude. For teacher self-efficacy, the difference between these two profiles was very small, despite being statistically significant.

### 4.3. The Mediating Role of Teacher Self-Efficacy in the Relationship Between Course Learning Experiences and Commitment to Teaching Across Profiles

**Figure 2:**
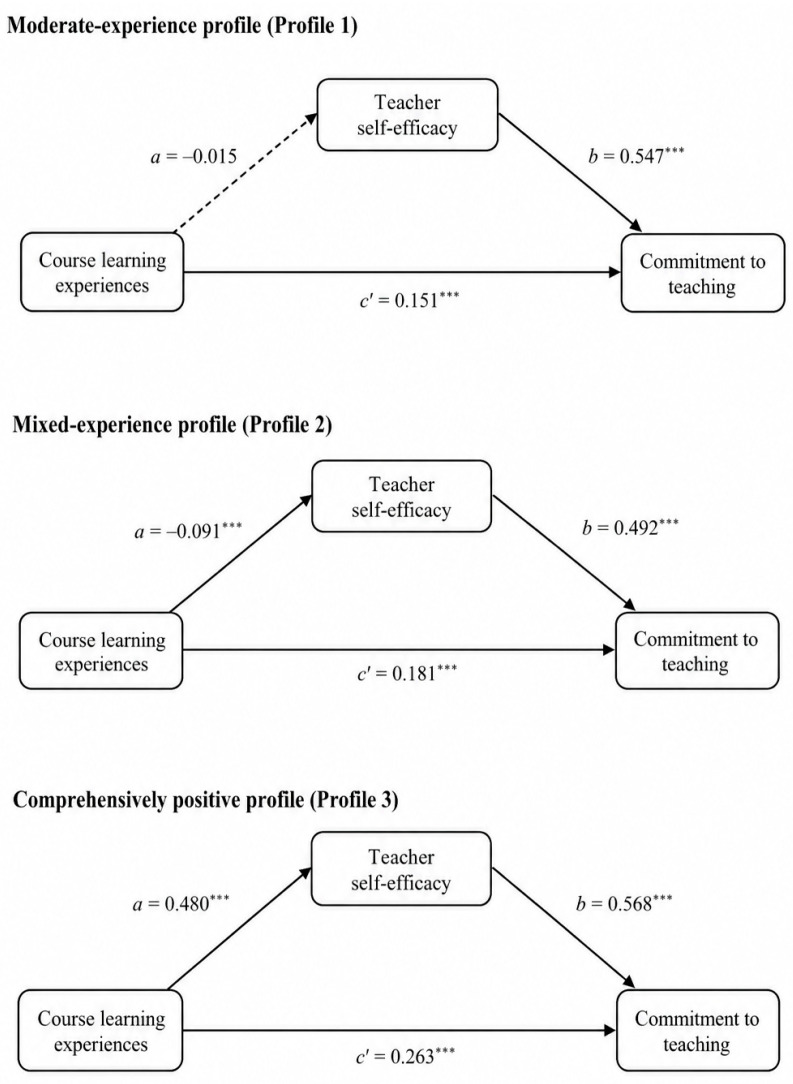
Profile-specific mediation pathways from course learning experiences to commitment to teaching through teacher self-efficacy. Note: Coefficients shown are unstandardized. Solid lines indicate significant paths; dashed lines indicate non-significant paths. Gender, grade level, and rural hukou status were included as covariates in all models but are omitted from the figure for clarity. *** *p* < 0.001.

As shown in Table 7 and Figure 2, the mediation patterns differed across the three latent profiles.

For the moderate-experience profile, course learning experiences were not significantly associated with teacher self-efficacy (B = −0.0150, 95% CI [−0.0585, 0.0285]), whereas teacher self-efficacy was significantly and positively associated with commitment to teaching (B = 0.5471, 95% CI [0.5276, 0.5666]). The total effect and direct effect of course learning experiences on commitment to teaching were both significant. However, the indirect effect through teacher self-efficacy was not significant (B = −0.0082, 95% bootstrap CI [−0.0373, 0.0201]), indicating that teacher self-efficacy did not mediate the relationship between course learning experiences and commitment to teaching in this profile.

For the mixed-experience profile, course learning experiences were negatively associated with teacher self-efficacy (B = −0.0910, 95% CI [−0.1300, −0.0521]), whereas teacher self-efficacy was positively associated with commitment to teaching (B = 0.4924, 95% CI [0.4761, 0.5088]). The total effect and direct effect of course learning experiences on commitment to teaching were both positive and significant. However, the indirect effect through teacher self-efficacy was significant and negative (B = −0.0448, 95% bootstrap CI [−0.0668, −0.0230]), indicating an inconsistent mediation pattern in this profile.

For the comprehensively positive profile, course learning experiences were positively associated with teacher self-efficacy (B = 0.4803, 95% CI [0.4540, 0.5066]), and teacher self-efficacy was positively associated with commitment to teaching (B = 0.5680, 95% CI [0.5452, 0.5907]). The total effect of course learning experiences on commitment to teaching was significant, and the direct effect remained significant after teacher self-efficacy was included. The indirect effect through teacher self-efficacy was also significant and positive (B = 0.2728, 95% bootstrap CI [0.2531, 0.2928]), indicating that teacher self-efficacy partially mediated the relationship between course learning experiences and commitment to teaching in this profile.

## 5. Discussion

Many factors may be associated with preservice teachers’ commitment to teaching. Drawing on the social learning theory of career decision making, this study focused on teacher education courses as a key learning context in which preservice teachers develop perceptions of teaching, evaluate their own teaching competence, and form commitment to the profession. Based on large-scale survey data from preservice teachers at local normal universities in China, this study identified three distinct profiles of learning experiences in teacher education courses: the moderate-experience profile, the mixed-experience profile, and the comprehensively positive profile. It further examined how teacher self-efficacy mediated the association between course learning experiences and commitment to teaching across these profiles.

By differentiating preservice teachers’ course learning experiences into distinct latent profiles, this study contributes to the application of the social learning theory of career decision making by showing that ”learning experiences” are not uniformly associated with career commitment. Rather, their effects depend on the specific configuration of teaching quality, course goal clarity, workload, and assessment practices. The findings also show that teacher self-efficacy functions differently across profiles, suggesting that preservice teachers’ career commitment is related not only to whether they have positive learning experiences, but also to how these experiences are organized and whether they foster a sense of teaching competence. In this sense, the study provides a person-centered understanding of the heterogeneity in preservice teachers’ course learning experiences and offers implications for teacher education course reform and quality improvement.

### 5.1. Three Distinct Profiles of Preservice Teachers’ Learning Experiences in Teacher Education Courses

Based on four dimensions—good teaching, clarity of course goals, appropriateness of workload, and appropriateness of assessment—the latent profile analysis identified three types of learning experiences in teacher education courses: the comprehensively positive profile, the mixed-experience profile, and the moderate-experience profile.

First, preservice teachers in the comprehensively positive profile scored relatively highly across all four dimensions, indicating the most favorable learning experiences. This suggests that they generally recognized the design of teacher education courses in terms of course goals, content, teaching, and assessment, and were able to develop positive learning attitudes supported by an appropriate workload and suitable feedback. This group illustrates the potential value of teacher education courses in promoting preservice teachers’ active engagement and professional identification, and provides an ideal reference point for course reform. Second, preservice teachers in the mixed-experience profile evaluated course goals and teaching quality positively, but expressed relatively strong dissatisfaction with workload and assessment practices. They generally perceived the current course workload as excessive and the assessment methods as placing too much emphasis on rote memorization, with insufficient connection to authentic teaching contexts. This finding reveals a key tension in teacher education course reform: although progress has been made in clarifying course goals and improving classroom teaching, excessive workload and a relatively narrow assessment model may undermine preservice teachers’ learning motivation and professional identification. This profile accounted for the largest proportion of the sample, highlighting the urgent need for teacher education course reform to respond to issues of workload reduction and assessment reform, so as to better balance learning quality with students’ learning experiences. Finally, preservice teachers in the moderate-experience profile reported scores close to the average across all dimensions, indicating an overall intermediate level of learning experience. The presence of this group suggests that, although these students did not express clear dissatisfaction, they had not fully experienced the motivational benefits of teacher education courses. This points to the need for further improvement in the attractiveness and effectiveness of teacher education courses.

Overall, this study found significant differentiation in preservice teachers’ perceptions of their learning experiences in teacher education courses. Notably, the mixed-experience profile accounted for the largest proportion of the sample, suggesting an urgent need to reform assignment design, academic workload, and assessment practices in teacher education courses. Future course reform should focus on reducing unreasonable academic workload and optimizing assessment practices, thereby better promoting positive learning experiences among preservice teachers.

### 5.2. High-Quality Teaching and Clear Course Goals Sustain Commitment, but Workload and Assessment Remain Critical Constraints

The three profiles differed significantly in commitment to teaching. Specifically, commitment to teaching was highest among preservice teachers in the comprehensively positive profile, followed by those in the mixed-experience profile, and lowest among those in the moderate-experience profile. This finding is broadly consistent with previous variable-centered studies showing that positive evaluations of teacher education coursework are often associated with stronger motivation to teach and lower uncertainty about career choice (Lee & Yuan, 2014; Tang et al., 2014). It suggests that preservice teachers’ learning experiences in teacher education courses are closely related to how they understand the value of teaching and whether they are willing to invest in the teaching profession over the long term.

Preservice teachers in the comprehensively positive profile reported the highest level of commitment to teaching. This result indicates that when teacher education courses are perceived as well-taught, clearly structured, appropriately assessed, and reasonably demanding, preservice teachers tend to report stronger professional commitment. Such experiences may help preservice teachers understand the meaning of teaching, develop positive expectations about their future professional roles, and strengthen their identification with the teaching profession. In this sense, teacher education courses are not merely vehicles for transmitting pedagogical knowledge and skills; they also provide important formative experiences through which preservice teachers gradually construct their professional orientation and commitment.

The mixed-experience profile provides a more complex picture. Preservice teachers in this profile expressed dissatisfaction with workload and assessment practices, yet their commitment to teaching remained higher than that of those in the moderate-experience profile. This suggests that favorable experiences with teaching quality and course goal clarity may help sustain commitment to teaching, even when other aspects of course learning are perceived negatively. Clear course goals can help preservice teachers understand what they are learning and why it matters, while high-quality teaching can make the connection between coursework and future teaching more visible. These positive elements may be associated with stronger commitment to teaching, possibly because they make the relevance and professional value of teacher education courses more visible.

However, the findings also show that positive teaching and clear goals are not sufficient to resolve all problems in course learning experiences. Assessment practices may have a dual effect on students’ course learning experiences and engagement: They can reinforce learning, but may also reduce motivation when perceived as overly rigid or unfair (Yin & Ke, 2017). In the present study, the mixed-experience profile accounted for the largest proportion of the sample, indicating that many preservice teachers recognized the value of teaching quality and clear course goals but remained dissatisfied with academic workload and assessment methods. This pattern reveals an important tension in teacher education course reform. On the one hand, teacher education courses may have made progress in clarifying learning goals and improving instructional quality. On the other hand, if assessment continues to rely heavily on rote memorization, repetitive assignments, or tasks weakly connected to authentic teaching contexts, preservice teachers may experience frustration and reduced learning motivation.

This tension can also be understood in relation to the broader educational context in China. Students are exposed to examination-oriented learning from an early age, and during senior secondary education they often devote substantial time to intensive study and examination preparation (Chen et al., 2020; Komatsu & Rappleye, 2018). Under the strong influence of the national college entrance examination, China’s competitive education system, together with high parental expectations, has intensified the academic pressure experienced by students (Liu et al., 2020). Such long-term exposure to academic pressure may make preservice teachers relatively accustomed to heavy workloads, thereby weakening the extent to which dissatisfaction with workload is directly associated with lower commitment to teaching. However, adaptation to pressure does not mean that such pressure is educationally desirable. After entering university, many students begin to reflect critically on exam-oriented learning traditions characterized by mechanical memorization rather than critical thinking. If teacher education courses continue to reproduce heavy workloads and exam-oriented assessment practices, preservice teachers may still experience dissatisfaction, even when their overall commitment to teaching remains relatively stable.

In addition, culture plays a key role in shaping values, social cognition, and decision-making processes (Alesina & Giuliano, 2015). In the Chinese teacher education context, preservice teachers may be more inclined to adapt to institutional expectations than to openly resist them. As authority figures, teacher educators may directly support preservice teachers’ commitment through clear goals and high-quality teaching. However, when workload and assessment are perceived as unreasonable, these positive experiences may not be sufficient to fully realize the developmental value of teacher education courses. This helps explain why the mixed-experience profile showed relatively strong commitment to teaching while still revealing clear problems in workload and assessment. By contrast, preservice teachers in the moderate-experience profile did not report strong dissatisfaction, but they also lacked sufficiently positive learning experiences that could foster stronger professional motivation and commitment.

Taken together, these findings suggest that the relationship between teacher education course experiences and commitment to teaching is not simply linear. High-quality teaching and clear course goals may help sustain preservice teachers’ commitment to teaching, whereas excessive workload and inappropriate assessment may constrain the developmental value of these courses. Therefore, teacher education course reform should not only improve instructional quality and clarify course objectives, but also pay close attention to whether workload and assessment practices are reasonable, meaningful, and connected to authentic teaching practice.

### 5.3. Teacher Self-Efficacy Shows Profile-Specific Indirect Associations Between Course Learning Experiences and Commitment to Teaching

The mediation results further reveal that teacher self-efficacy is not merely an independent correlate of commitment to teaching, but a key psychological condition through which course learning experiences may be indirectly associated with professional commitment. This finding is broadly consistent with the social learning theory of career decision making, which emphasizes that individuals’ career choices are shaped through learning experiences, self-observation, self-evaluation, and perceived competence (Krumboltz et al., 1976; Krumboltz & Vosvick, 1996). It also echoes Rots and Aelterman’s (2008, 2009) model, which suggests that teacher education experiences influence preservice teachers’ career choices not only directly but also by shaping their teacher self-efficacy and affective orientations toward the teaching profession.

However, the present study goes beyond prior variable-centered findings by showing that the mediating role of teacher self-efficacy differs across profiles of course learning experiences. In the comprehensively positive profile, teacher self-efficacy served as a significant positive mediator between course learning experiences and commitment to teaching. This suggests that when preservice teachers experience teacher education courses as coherent, supportive, well-taught, appropriately assessed, and reasonably demanding, these experiences were associated with stronger confidence in their teaching capabilities and higher commitment to teaching. This finding is consistent with previous studies showing that positive teacher education experiences, supportive learning environments, and high-quality teaching are associated with stronger teaching motivation and professional identity among preservice teachers (Lee & Yuan, 2014; Tang et al., 2014). It also supports the view that teacher self-efficacy is developed through meaningful learning experiences and opportunities to perceive oneself as capable of meeting professional demands (Tschannen-Moran & Woolfolk Hoy, 2001; Tschannen-Moran & McMaster, 2009).

By contrast, the mixed-experience profile showed a significant negative indirect effect through teacher self-efficacy, suggesting an inconsistent mediation pattern. This counterintuitive pattern may reflect the particular configuration of this profile, in which positive perceptions of teaching quality and course goal clarity coexisted with negative perceptions of workload and assessment. Within this profile, the overall course learning experience score may therefore capture a complex balance among dimensions rather than a uniformly positive learning condition. Thus, the negative indirect effect should be interpreted as a profile-specific inconsistent mediation pattern, rather than as evidence that better course learning experiences generally reduce teacher self-efficacy. This result helps explain why positive course elements do not necessarily translate smoothly into stronger professional commitment. Prior research has shown that assessment practices can both support and undermine student engagement, depending on whether they are perceived as meaningful, fair, and developmentally appropriate (Yin & Ke, 2017). Similarly, while appropriately challenging coursework may strengthen commitment to teaching (Sinclair, 2008), excessive workload or poorly aligned assessment may reduce motivation and professional confidence (Chambers et al., 2010; Roness & Smith, 2010). Therefore, in the mixed-experience profile, positive perceptions of teaching and course goals may have helped sustain commitment to teaching, whereas dissatisfaction with workload and assessment may have constrained the extent to which course learning experiences were associated with teacher self-efficacy.

For the moderate-experience profile, teacher self-efficacy did not significantly mediate the association between course learning experiences and commitment to teaching. This finding suggests that merely “acceptable” course experiences may be insufficient to activate teacher self-efficacy as a meaningful psychological pathway. Preservice teachers in this profile did not report strong dissatisfaction, but they also lacked highly positive learning experiences that could help them build confidence, professional identification, and a stronger sense of competence. This finding is important because it indicates that the absence of negative course experiences does not necessarily mean that teacher education courses are developmentally powerful. To foster commitment to teaching, teacher education courses may need to provide experiences that are not only adequate, but also sufficiently engaging, supportive, and professionally meaningful.

Taken together, these findings refine existing understandings of the relationship between teacher self-efficacy and commitment to teaching. Previous studies have consistently shown that teacher self-efficacy is positively associated with commitment to teaching (Coladarci, 1992; Ware & Kitsantas, 2007), and Chesnut and Burley’s (2015) meta-analysis further confirmed the importance of self-efficacy in predicting commitment to the teaching profession. The present study adds to this literature by showing that teacher self-efficacy does not operate in the same way for all preservice teachers. Rather, its mediating role depends on the configuration of course learning experiences. When teacher education courses provide coherent and positive experiences, teacher self-efficacy becomes a pathway through which course learning experiences strengthen commitment to teaching. When course experiences are mixed or merely moderate, this pathway may be weakened or fail to emerge.

Thus, the findings suggest that teacher education programs should not assume that course participation itself will naturally enhance preservice teachers’ commitment to teaching. What matters is whether course learning experiences help preservice teachers develop a stronger belief that they are capable of becoming competent teachers. From this perspective, teacher self-efficacy serves as a crucial bridge between what preservice teachers experience in teacher education courses and whether they develop a firm commitment to entering and remaining in the teaching profession.

## 6. Practical Implications and Recommendations

### 6.1. Optimizing Teacher Education Course Design to Address the Differentiated Needs of Preservice Teachers Across Profiles

The three profiles identified through latent profile analysis differed significantly in preservice teachers’ experiences of teacher educators’ teaching, course goals, academic workload, and assessment practices. Preservice teachers in the comprehensively positive profile reported the highest levels of teacher self-efficacy and commitment to teaching. This finding suggests that teacher education course reform should include regular monitoring of preservice teachers’ course learning experiences. On this basis, differentiated instructional design should be implemented through profile-based classification and targeted support tailored to students’ diverse needs. For example, preservice teachers in the comprehensively positive profile reported generally favorable overall experiences in teacher education courses, although there remains room for further improvement. Building on high-quality teaching and clearly articulated course goals, teacher education programs should further expand interdisciplinary course offerings and adopt more innovative pedagogical approaches to help these preservice teachers deepen their professional identity and enhance their capacity for educational innovation. For example, teacher education programs could introduce course modules that integrate educational technology with subject teaching to cultivate preservice teachers’ digital teaching competence, and offer courses in educational research methods to encourage their participation in research projects and enhance their academic literacy. For preservice teachers in the mixed-experience profile, who reported more negative experiences with academic workload and assessment practices, course reform should pay particular attention to reducing unreasonable workload and improving assessment design. Therefore, course workload should be appropriately adjusted, and academic support and advising services should be provided to help preservice teachers cope with academic pressure. For example, academic tutoring centers could be established to provide regular one-on-one guidance, and academic advising, workload coordination, and formative feedback mechanisms should be provided to help preservice teachers cope with academic stress. Preservice teachers in the moderate-experience profile require particular attention from teacher educators. For this group, the key is to stimulate their learning motivation by clarifying course goals, increasing classroom interaction and practice-oriented components, and designing learning tasks that are both appropriately challenging and engaging, thereby activating their intrinsic motivation. For example, project-based learning can be adopted to enable students to acquire knowledge through practice; group-based competitions can be designed to stimulate learning enthusiasm; and reflective teaching journals, micro-teaching tasks, and case-based lesson design activities can be developed to enhance their intrinsic motivation. In sum, teacher education courses should incorporate differentiated interventions to help preservice teachers across different profiles develop positive learning experiences, thereby improving the overall quality of preservice teacher preparation.

### 6.2. Reforming Course Assessment Practices to Alleviate Learning Pressure and Enhance Learning Engagement

A substantial proportion of preservice teachers, especially those in the mixed-experience profile, reported relatively negative experiences with assessment practices and academic workload in teacher education courses. These dimensions therefore represent important areas for course reform. Therefore, optimizing assessment practices should serve as a key entry point for teacher education course reform. On the one hand, teacher education courses should gradually reduce their reliance on single examination scores and rote memorization, and move toward more diversified and developmental forms of assessment. For example, where appropriate, teacher educators can use digital tools to support formative assessment by documenting students’ learning processes and progress. They can also implement performance-based assessment, evaluating students’ competencies through classroom performance, group discussions, and other interactive activities. In addition, project-based assessment can be adopted to encourage students to complete integrative projects and demonstrate their comprehensive abilities. On the other hand, course workload should be kept at a reasonable level, and the accumulation of mechanical assignments should be avoided. Instead, excessive repetitive assessments should be replaced with collaborative group work, inquiry-based tasks, and interdisciplinary projects. At the same time, teacher educators should communicate course goals and learning expectations more clearly during instruction, helping students understand the rationale behind course design and thereby reducing anxiety and resistance caused by perceived workload. For example, course orientation sessions can be held at the beginning of each semester to clarify course objectives and assessment requirements. Teacher educators should also communicate with students regularly to identify their learning difficulties and provide timely support. These measures may help preservice teachers accumulate positive learning experiences in an environment characterized by reasonable academic pressure and appropriate assessment, thereby supporting their professional identity and commitment to teaching.

### 6.3. Enhancing Preservice Teachers’ Commitment to Teaching by Strengthening Teacher Self-Efficacy

Because teacher self-efficacy was consistently associated with commitment to teaching across profiles, teacher education courses should provide more practice-oriented and mastery-supportive learning opportunities. First, student-centered pedagogical strategies should be adopted. Teacher education courses should incorporate inquiry-based activities, case analysis, and collaborative learning, while placing greater emphasis on situated teaching and learning. Second, learning support systems for preservice teachers should be further strengthened. Teacher education programs may explore mentoring systems, peer support, and professional learning communities to provide preservice teachers with role modeling and emotional support. In addition, professional learning community activities can be organized regularly to enable preservice teachers to share teaching experiences, reflections, and practical insights. Through these measures, preservice teachers can strengthen their identification with the teaching profession and enhance their sense of teacher self-efficacy, thereby further promoting their commitment to teaching.

## 7. Limitations and Future Perspectives

Several limitations should be acknowledged. First, although this study used a large-scale sample of preservice teachers from local normal universities in China, the data were cross-sectional, which prevents direct causal inference. Future research could employ longitudinal designs to further examine how preservice teachers’ learning experiences in teacher education courses influence the development of their teacher self-efficacy and commitment to teaching over time.

Second, this study mainly relied on self-report questionnaire data. Although the measures demonstrated acceptable reliability and validity, self-reported data may still be subject to social desirability bias and individual response bias. Future studies could combine survey data with interviews, classroom observations, and institution-level data to provide a more comprehensive understanding of preservice teachers’ course learning experiences and professional development.

Third, this study focused on local normal universities in China. Although these institutions play an important role in the Chinese teacher education system, the extent to which the findings can be generalized to other types of teacher education institutions or different national contexts requires further examination. Future research could conduct comparative studies across institutional types, regions, and cultural contexts to investigate whether similar profiles of course learning experiences exist under different educational systems and cultural conditions.

Fourth, this study examined the mediating role of teacher self-efficacy in the association between course learning experiences and commitment to teaching. Although this analysis helps reveal one important psychological pathway linking course experiences to professional commitment, the relationship between course learning experiences and commitment to teaching may be shaped by multiple factors. Future research could incorporate additional mediating and moderating variables, such as professional identity, teaching motivation, perceived career prospects, institutional support, and practicum experiences, to develop a more nuanced understanding of this relationship. Moreover, future studies could further distinguish among more specific dimensions of course learning experiences and examine how different combinations of course goals, teaching quality, workload, assessment practices, feedback, and practical relevance jointly influence preservice teachers’ commitment to teaching.

## Figures and Tables

**Table 1:** The characteristics of the sample.

Mean (Standard Deviation) or Proportion		
	Sample (N)	Proportion (%)
Year		
2nd year	13,951	53.4
3rd year	12,159	46.6
Gender		
Male	6052	23.2
Female	20,058	76.8
Residential registration (*Hukou*)		
Rural	17,936	68.7
Urban	8174	31.3
Residence		
Villages	10,611	40.6
Towns	8822	33.8
Cities	6677	25.6
Total	26,110	100

**Table 2:** Descriptive Statistics for the Study Variables.

Variable	Min	Max	M	SD
Good Teaching (GT)	1	5	3.825	0.773
Clear course goals (CCG)	1	5	3.658	0.627
Appropriate Assessment (AA)	1	5	2.861	0.964
Appropriate Workload (AW)	1	5	2.92	1.008
Course learning experiences (CLE)	1	5	3.316	0.579
Teacher self-efficacy (TSE)	1	5	3.915	0.618
Commitment to teaching (CT)	1	5	3.66	0.643

Note. N = 26,110. M = mean; SD = standard deviation. All variables were measured on a five-point Likert scale.

**Table 3:** Fit Indices for the Latent Profile Models.

Profile	LL	AIC	BIC	aBIC	pLMR	pBLRT	Entropy
2-profile	−121,568.8	243,163.6	243,269.8	243,228.5	0.000	0.000	0.743
3-profile	−115,422.9	230,881.7	231,028.8	230,971.6	0.000	0.000	0.752
4-profile	−111,936.1	223,918.2	224,106.1	224,033.0	0.000	0.000	0.822
5-profile	−109,660.1	219,376.1	219,604.9	219,515.9	0.000	0.000	0.798
6-profile	−105,728.5	211,523.0	211,792.6	211,687.7	0.000	0.000	0.872

**Table 4:** Mean Scores of Course Learning Experience Dimensions Across the Three Latent Profiles.

Profiles	N	% of Sample	GT	CCG	AA	AW
1	6640	25.4	2.92	3.00	2.94	2.91
2	10,674	40.9	4.09	3.66	2.13	2.21
3	8796	33.7	4.21	4.17	3.69	3.79

**Table 5:** Between-Profile Differences in Preservice Teachers’ Commitment to Teaching and Teacher Self-Efficacy.

Variable	Profile	Mean	S.E.	Group Differences (Profile k-Profile k + i, i = 0, 1, 2)			Overall Chi-Square Test
				Profile 1	Profile 2	Profile 3	
Commitment to Teaching	1	3.22	0.009	-			3551.944 ***
	2	3.70	0.006	−0.48 ***	-		
	3	3.95	0.007	−0.73 ***	−0.25 ***	-	
Self-Efficacy	1	3.42	0.010	-			4582.101 ***
	2	4.08	0.006	−0.66 ***	-		
	3	4.11	0.006	−0.69 ***	−0.03 ***	-	

Note. *** *p* < 0.001; the degrees of freedom for the overall chi-square test were df = 2.

**Table 6:** Pairwise Cohen’s d Effect Sizes for Differences in Commitment to Teaching and Teacher Self-Efficacy Across Profiles.

	Commitment to Teaching			Self-Efficacy		
	Profile 1	Profile 2	Profile 3	Profile 1	Profile 2	Profile 3
Profile 1	—	−0.821	−1.143	—	−1.111	−1.173
Profile 2	0.821	—	−0.436	1.111	—	−0.057
Profile 3	1.143	0.436	—	1.173	0.057	—

Note. Positive values indicate that the row profile scored higher than the column profile, whereas negative values indicate that the row profile scored lower than the column profile.

**Table 7:** Mediation Analysis of Teacher Self-Efficacy in the Relationship Between Course Learning Experiences and Commitment to Teaching Across Profiles.

	Profile 1 (n = 6640)		Profile 2 (n = 10,674)		Profile 3 (n = 8796)	
Effect	B	95% CI	B	95% CI	B	95% CI
Path a: Course learning experiences → Teacher self-efficacy	−0.0150	[−0.0585, 0.0285]	−0.0910 ***	[−0.1300, −0.0521]	0.4803 ***	[0.4540, 0.5066]
Path b: Teacher self-efficacy → Commitment to teaching	0.5471 ***	[0.5276, 0.5666]	0.4924 ***	[0.4761, 0.5088]	0.5680 ***	[0.5452, 0.5907]
Total effect, c: Course learning experiences → Commitment to teaching	0.1427 ***	[0.1001, 0.1852]	0.1363 ***	[0.0977, 0.1749]	0.5359 ***	[0.5036, 0.5682]
Direct effect, c′: Course learning experiences → Commitment to teaching	0.1509 ***	[0.1156, 0.1861]	0.1811 ***	[0.1477, 0.2147]	0.2631 ***	[0.2325, 0.2938]
Indirect effect through teacher self-efficacy	−0.0082	[−0.0373, 0.0201]	−0.0448 ***	[−0.0668, −0.0230]	0.2728 ***	[0.2531, 0.2928]
R^2^ for mediator model	0.0052		0.0165		0.1463	
R^2^ for outcome model	0.3207		0.2549		0.3081	

Note. Values in brackets are 95% confidence intervals. For indirect effects, the values in brackets are bootstrap confidence intervals based on 5000 resamples. Gender, grade level, and rural hukou status were included as covariates. R^2^ values refer to the explained variance of the mediator and outcome models, respectively. *** *p* < 0.001.

## Data Availability

The data presented in this study are available upon request from the corresponding author. The data are not publicly available due to confidentiality and ethics reasons.
